# Assessment of counselling for acute diarrhoea in German pharmacies: a simulated patient study

**DOI:** 10.1111/ijpp.12405

**Published:** 2017-09-19

**Authors:** Bernhard Langer, Elisa Bull, Tina Burgsthaler, Julia Glawe, Monique Schwobeda, Karen Simon

**Affiliations:** ^1^ Faculty of Health Nursing Administration University of Applied Sciences Neubrandenburg Neubrandenburg Germany

**Keywords:** acute diarrhoea, community pharmacy, counselling behaviour, non‐prescription medicines, simulated patient

## Abstract

**Objectives:**

The aim of this study was to assess the quality of counselling provided for acute diarrhoea and to evaluate the role of the patient's approach and different user groups in determining the outcome of counselling.

**Methods:**

The simulated patient methodology was used in all 21 community pharmacies in a north‐eastern German city. Four different scenarios related to self‐medication of acute diarrhoea were developed and used in all the pharmacies (a total of 84 visits). The assessment form, completed immediately postvisit by the simulated patient, included 9 objective items scored using dichotomous scales to produce a scale from 0 to 9. After evaluating the data, every pharmacy received individual performance feedback to encourage behavioural changes and improve the quality of the counselling provided.

**Key findings:**

Overall, the quality of counselling was poor (mean score of 3.3/9 (37%)). The most common information provided was about dosage (87% of interactions), while the least common information given was about side effects (4% of interactions). The main effect was seen when comparing the product and symptom requests (*F*(1,60) = 24.748, *P* < 0.001, ω_p_^2^ = 0.277). There was no effect resulting from different user groups (*F*(1,28) = 0.237, *P* = 0.630, ω_p_^2^ = −0.026) and no interaction between the type of request and different user groups (*F*(1,28) = 3.395, *P* = 0.076, ω_p_^2^ = 0.073).

**Conclusions:**

This study highlighted the current deficits in appropriate counselling provided by community pharmacies in Germany.

## Introduction

Worldwide, the number of medicinal products that can be obtained over the counter, that is, purchased in pharmacies without a medical prescription, is growing.^1^ The reasons for this are that medicinal products that previously required a prescription are increasingly being removed from the list of prescription‐only medicinal products (OTC switch) along with patient preferences for taking greater responsibility for their own health.^2^ In Germany, for example, 72% of citizens indicate in a survey conducted by the Federal Union of German Associations of Pharmacists (ABDA) that for minor health issues they would first go to a pharmacy.^3^

Of the approximately 100 000 medicinal products with marketing authorisation in Germany, about half belong to the over‐the‐counter medicinal products market segment.^4^ Even when measuring the share of sales in package units, this market segment still represents about half the share.^5^ Because these preparations are largely used for self‐medication, expert advice from the pharmacy is essential. Pharmacies can help to prevent risks resulting from improper use or at least significantly reduce these risks, because employees in the pharmacies should have extensive knowledge about the medications in terms of their effects, side effects and use.

Good advice is not only helpful for the customer or patient, but also plays a crucial role in competition between pharmacies. The freedom to establish pharmacies on the one hand and the increasing competition with mail‐order pharmacies in recent years on the other means that there is a brisk competitive environment in many areas of Germany. A good advisory service in this context is an important means of gaining a competitive edge.

Various studies available for Germany show that the quality of the advice provided to customers by pharmacies has considerable room for improvement.^6^, ^7^, ^8^, ^9^, ^10^ However, these studies refer to indications other than acute diarrhoea, meaning that publicly accessible studies on the quality of advice provided by pharmacies in Germany for this indication are not yet available. Acute diarrhoea is also quite an important indication because it is one of the most common medical conditions in Germany. About 41 million cases of acute diarrhoea can be assumed each year, about two‐thirds of which are treated without a medical consultation.^11^ If diarrhoea is treated by self‐medicating, those affected are commonly dependent on the advice by the pharmacy because acute diarrhoea can be a symptom of a wide range of medical conditions. It is therefore also necessary to query how the symptoms have progressed to take into account the duration, concomitant symptoms and the severity and, where necessary, to recommend the patient seek medical advice.^12^

The primary aim of this study was to investigate the counselling practices of community pharmacies provided for acute diarrhoea using simulated patients. The secondary objective was to evaluate the role of the patient's approach and different user groups in determining the outcome of counselling.

## Methods

### Design

The study used the ‘simulated patient’ method to determine the quality of advice provided by the pharmacies being assessed, a procedure that is a type of mystery shopping. This approach was used to assess the quality of services provided and is a type of participatory observation. The test buyers simulated an apparently real situation to participate in a service provision process. This was subsequently evaluated by the test buyers to reveal any possible deficiencies.^13^ The method is established internationally to determine the quality of advice provided by pharmacies.^14^, ^15^, ^16^, ^17^, ^18^, ^19^

Five female Master students of a German University (University of Applied Sciences Neubrandenburg, Faculty of Health, Nursing, Administration) were selected to be the Simulated Patients. The selection process was based on their participation in a student research project in their first year of graduate studies in Health Sciences.

### Setting and participation

The test purchases were made between the start of May 2014 and the end of July 2014 in the city of Neubrandenburg (about 63 000 residents; located in the state of Mecklenburg‐Western Pomerania), which has 21 pharmacies in the city region.^20^ Each pharmacy was visited once using four different test scenarios, giving a total of 84 test purchases (4 scenarios × 1 visit × 21 pharmacies). When carrying out the individual test purchases, there was no differentiation made regarding the education of the pharmacy personnel providing the advice. A total of €375.87 was required to carry out these test purchases, a sum that was drawn from the resources of the primary author.

### Outcomes and outcome measurements

According to work regulations for pharmacies, pharmacies in Germany are obliged to implement a quality management system that is intended to ensure a comprehensive advisory service.^21^ In this context, the German Federal Chamber of Pharmacists drafts a range of guidelines and working aids such as the document ‘Information and advisory service as part of self‐medication using the example of self‐diagnosis of diarrhoea’.^12^ This formed the basis for the assessment form used in this study that is shown in Table 1. The assessment form only included 9 objective items that refer to the pharmacological advisory service in order to avoid a subjective evaluation and thus any latitude in the assessments made by the test buyers (such as the friendliness of the customer contact).^22^ For this reason, the individual items were only completed using dichotomous scales (closed yes/no questions). Weighting of the individual items was omitted because this in turn would depend on subjective considerations. To avoid influencing the quality of the advisory service provided by the pharmacies being tested and thus possibly a situation that no longer reflects reality (Hawthorne effect), the test purchases were covert; that is, they were conducted without informing the particular pharmacy beforehand, using a similar approach to some other international studies.^14^

**Table 1:** Assessment form

Variables	Coding	
	Not discussed	Discussed
For whom is the medicinal product?	0	1
How long have the symptoms been present?	0	1
How often do the symptoms occur?	0	1
What other symptoms are present?	0	1
Have the symptoms already been clarified by a doctor?	0	1
Are other diseases present or what medicinal products are currently/regularly used?	0	1
How does the medication have to be used?	0	1
How long can/must the medication be taken?	0	1
What side effects may occur?	0	1

Because there is clear evidence in the international literature of differences in the reporting quality between symptom‐based and medication‐based requests,^1^, ^6^, ^23^, ^24^ this study aimed to determine whether these differences also apply to the indication ‘acute diarrhoea’ in addition to determining pharmacies’ compliance with the standard of advice required. For this reason, two of the scenarios were designed as medication based (test scenarios 1 and 3) and the other two were symptom based (test scenarios 2 and 4). In the first and third scenarios, the test buyers made a medication‐based enquiry about the active substance loperamide, which is most commonly provided by pharmacies in Germany for self‐medication of acute diarrhoea and is a component of various over‐the‐counter medicinal products.^25^ In contrast, in the second and fourth scenarios, the test buyers requested a medication to treat acute diarrhoea, making a symptom‐based enquiry.

We also intended to test whether the quality of the advisory service provided by pharmacies differed depending on whether the preparation requested was for an older person with underlying diseases or for a younger person with no underlying diseases. For this reason, the test buyers in the first and second scenarios made the request on behalf of their 74‐year‐old grandmother (with diabetes mellitus and hypertension as the underlying diseases), while in the third and fourth scenarios, the request was for a 30‐year‐old partner (with no underlying diseases). Otherwise, the information given to the pharmacy sales personnel in response to their questions was identical for each of the four test purchases (see Table 2).

**Table 2:** Test scenarios

*Scenario 1*
The test buyer enters the pharmacy and asks for a pack of loperamide. If the pharmacist offers a substitute preparation, the test buyer is willing to accept it. This is regardless of whether a medication with a different active substance or a homoeopathic preparation is offered
If the pharmacist asks, the following information is supplied:
Preparation is for the test buyer's 74‐year‐old grandmother Acute diarrhoea present for 24 h and has occurred several times up to now The grandmother's fluid intake is adequate, no vomiting, no blood in the stool, no fever Has not yet visited a doctor Underlying conditions: Diabetes and high blood pressure
*Scenario 2*
The test buyer enters the pharmacy and asks for a preparation to treat diarrhoea. The test buyer does not have any particular product in mind
If the pharmacist asks, the following information is supplied:
Preparation is for the test buyer's 74‐year‐old grandmother Acute diarrhoea present for 24 h and has occurred several times up to now The grandmother's fluid intake is adequate, no vomiting, no blood in the stool, no fever Has not yet visited a doctor Underlying conditions: Diabetes and high blood pressure
*Scenario 3*
The test buyer enters the pharmacy and asks for a pack of loperamide. If the pharmacist offers a substitute preparation, the test buyer is willing to accept it. This is regardless of whether a medication with a different active substance or a homoeopathic preparation is offered
If the pharmacist asks, the following information is supplied:
Preparation is the test buyer's 30‐year‐old partner Acute diarrhoea present for 24 h and has occurred several times up to now The partner's fluid intake is adequate, no vomiting, no blood in the stool, no fever Has not yet visited a doctor No underlying conditions
*Scenario 4*
The test buyer enters the pharmacy and asks for a preparation to treat diarrhoea. The test buyer does not have any particular product in mind
If the pharmacist asks, the following information is supplied:
Preparation is the test buyer's 30‐year‐old partner Acute diarrhoea present for 24 h and has occurred several times up to now The partner's fluid intake is adequate, no vomiting, no blood in the stool, no fever Has not yet visited a doctor No underlying conditions

### Data collection

The test buys in each scenario were made at 2‐week intervals. There was 1 week between each of the individual scenarios (with 21 test purchases each). The pharmacies to be tested were allocated randomly to the particular test buyers in the first step. As a result, each test buyer was allocated a total of 16–18 test purchases across all the scenarios. After the allocation but before the visits, we subsequently checked and ensured that none of the pharmacies were visited multiple times by any one test buyer to prevent the risk of the test buyers being exposed. This produced an overview of which test buyers visited which pharmacy using which scenario at what time. Before data collection was started, each test buyer carried out four pretests outside Neubrandenburg for each of the four scenarios. The functionality of the assessment form and the four test scenarios was confirmed in the process.

For data privacy reasons, audio recordings were not adopted. However, the assessment forms were completed by the test buyers immediately after visiting the pharmacies so that recall bias by the test buyers could be minimised.

After evaluating the data collection, each pharmacy was given individual performance feedback in which the particular pharmacy was assessed relative to the other anonymised competitors in the city of Neubrandenburg using benchmarking. This gave the pharmacies information about their competitive position so that ideally the pharmacies visited could implement appropriate optimisation processes based on this information with the aim of sustainably improving the quality of their advisory service.

### Data management and analysis

The statistical software program SPSS 22 was used to record and analyse the data. Items were scored dichotomously (0 = variable not met, 1 = variable met). The data obtained were initially analysed using descriptive statistics. To investigate whether there were significant effects on the counselling quality due to the type of request (product versus symptom‐based request), different user groups (74‐year‐old grandmother with diabetes mellitus and hypertension versus 30‐year‐old partner with no underlying diseases) and the interaction between the type of request and different types of user groups, a mixed model ANOVA was carried out. In this model, each community pharmacy served as a level of a random factor while request and user group both served as fixed factors. Results were considered statistically significant if *P* values were less than 0.05.

### Ethical approval

Following the ‘Guideline for the use of mystery research in market and social research’,^26^ the information obtained was recorded so that the pharmacists involved could not be identified and the results were reported anonymously. The study was approved retrospectively by the local University ethics committee, which was established after the study had commenced.

## Results

All 84 planned simulated patient visits were completed. At the level of the individual variables, assessing 21 pharmacies produced a mean score of 7.7 of a possible 21 points (37%) across all test scenarios. The mean scores varied greatly between 0.8 points (4%) for the advice provided about side effects and 18.3 points (87%) for the advice provided about dosage. The advice provided about duration and the question ‘for whom’ also achieved relatively high mean scores of 14.0 points (67%) and 13.0 points (62%), respectively, whereas, for example, the questions asked about ‘diseases’ and ‘clarification by a doctor’ achieved relatively low mean scores of 1.5 points (7%) and 3.8 points (18%), respectively (see Table 3).

**Table 3:** Assessment at variable level

	Scenario 1	Scenario 2	Scenario 3	Scenario 4	Mean	Percentage
For whom?	5	16	12	19	13.0	62
For how long?	4	14	5	9	8.0	38
How often?	0	9	3	5	4.3	20
Concomitant symptoms?	0	9	6	8	5.8	27
Clarification by a doctor?	1	8	2	4	3.8	18
Diseases?	0	3	0	3	1.5	7
Dosage	17	21	16	19	18.3	87
Duration	15	15	9	17	14.0	67
Side effects	0	0	2	1	0.8	4
Mean	4.7	10.6	6.1	9.4	7.7	37

At the level of the pharmacy, across the total of 9 variables on the assessment form and the dichotomous coding for each test scenario, each pharmacy obtained a result between 0 points (minimum) and 9 points (maximum). Across all four test scenarios, a mean score of 3.3 of a possible 9 points (37%) was achieved. The mean scores achieved varied greatly between 1.0 points (11%) and 5.0 points (56%) (see Table 4). The main effect was seen when comparing product and symptom requests (*F*(1,60) = 24.748, *P* < 0.001, ω_p_^2^ = 0.277). Symptom‐based requests had a mean score of 4.3 of a possible 9 points (48%) compared to direct product requests with a mean score of 2.3 of a possible 9 points (26%). For the medication‐based enquiries, the advice received was accordingly considerably worse than for scenarios that did not involve a direct request for the active substance loperamide.

**Table 4:** Assessment at pharmacy level

	Scenario 1	Scenario 2	Scenario 3	Scenario 4	Mean	Percentage
Pharmacy 1	3	4	6	4	4.3	47
Pharmacy 2	3	7	3	7	5.0	56
Pharmacy 3	0	4	2	8	3.5	39
Pharmacy 4	2	2	6	2	3.0	33
Pharmacy 5	2	5	6	4	4.3	47
Pharmacy 6	4	5	0	7	4.0	44
Pharmacy 7	1	5	1	6	3.3	36
Pharmacy 8	4	3	2	6	3.8	42
Pharmacy 9	1	1	1	1	1.0	11
Pharmacy 10	2	5	1	3	2.8	31
Pharmacy 11	3	6	3	4	4.0	44
Pharmacy 12	2	5	2	1	2.5	28
Pharmacy 13	2	5	6	4	4.3	47
Pharmacy 14	3	6	3	6	4.5	50
Pharmacy 15	2	6	0	3	2.8	31
Pharmacy 16	2	7	0	4	3.3	36
Pharmacy 17	0	1	1	4	1.5	17
Pharmacy 18	0	7	2	2	2.8	31
Pharmacy 19	2	4	0	1	1.8	19
Pharmacy 20	0	1	4	5	2.5	28
Pharmacy 21	4	6	6	3	4.8	53
Mean	2.0	4.5	2.6	4.0	3.3	37

In terms of the question of whether the advice provided differed for the different user groups, in all four scenarios those pharmacies in which the variable ‘For whom is the medication?’ had a value of zero were first extracted. This enabled the data to be filtered for those pharmacies that knew who the particular user of the medication would be (see supporting information Data S1 and Table S1).

There was no effect associated with the user group (*F*(1,28) = 0.237, *P* = 0.630, ω_p_^2^ = −0.026). Requests for a 74‐year‐old grandmother with underlying diseases had a mean score of 5.0 of a possible 9 points (56%), whereas requests for a 30‐year‐old partner with no underlying diseases had a mean score of 4.2 of a possible 9 points (47%).

There was no interaction between the type of request and the different user groups (*F*(1,28) = 3.395, *P* = 0.076, ω_p_^2^ = 0.073). The symptom‐based requests for the grandmother had a mean score of 5.4 of a possible 9 points (60%) compared to symptom‐based requests for the partner with a mean score of 4.3 of a possible 9 points (48%). The medication‐based requests for the grandmother achieved a mean score of 3.6 of a possible 9 points (40%) compared to medication‐based requests for the partner with a mean score of 3.9 of a possible 9 points (43%).

## Discussion

The results of the present study made clear that the quality of advice provided for acute diarrhoea in adults in a north‐eastern German city was poor. The most common information provided was about dosage, while the least common information given was about side effects. Significant differences were seen when comparing the product and symptom requests, but there was no effect resulting from different user groups and no interaction between the type of request and different user groups.

This study was limited by the fact that it only investigated the quality of advice provided by pharmacies in a medium‐sized regional city in a particular region in Germany. It would therefore be useful if future studies are (ideally) based on an adequately large, randomised sample of pharmacies drawn from across Germany. It must also be noted as a further limitation that only four test purchases were carried out in each pharmacy. It would therefore be plausible to increase the number of test purchases per pharmacy for future studies.

Because it cannot be ruled out that the test results varied depending on the particular test buyers used, it is also possible that evaluations by other test buyers would lead to different results. On the other hand, using only objective items avoided possible leeway in the assessments made by the test buyers. This may have led to an increased reliability, but at the expense of validity. For example, pharmacy staff can ask questions in a great variety of quality, and reducing these to binary items conceals that real variability.

When carrying out the individual test purchases, there was no differentiation made regarding the education of the pharmacy personnel providing the advice. Therefore, it was not possible to determine whether there are significant differences in the advice provided by pharmacists and pharmaceutical technical assistants. Future studies should take this aspect into account, especially as there are contradictory study results in the international literature in this regard.^7^, ^23^, ^27^, ^28^, ^29^

In this study, clear potential was found for almost all Neubrandenburg pharmacies to optimise the quality of advice provided for acute diarrhoea in adults. Similar problems are also described in a recent Turkish study.^27^

Although the advice dispensed was only tested for the indication acute diarrhoea, other scientific studies in Germany of other indications (such as headaches) reveal that there is considerable room for improvement.^6^, ^7^ Stiftung Warentest, a consumer organisation in Germany, also conducts test purchases at regular intervals and identifies a rather poor quality advisory service.^8^, ^10^ This is in no way a problem limited to Germany because such deficiencies in the advice provided in pharmacies are described many times in the international literature.^23^, ^29^, ^30^, ^31^, ^32^, ^33^, ^34^, ^35^, ^36^, ^37^, ^38^ On the other hand, there are also a few examples internationally of in part appropriate advisory services provided by pharmacies.^39^, ^40^

Depending on the particular criteria investigated, there were clear differences apparent in the quality of the advice provided. While, for example, barely any information was provided for the criterion ‘side effects’, pharmacies often provided information about the dosage of the medication. Similar differences are also seen in the international literature.^6^, ^7^, ^16^, ^28^, ^30^, ^31^, ^41^, ^42^

What was noticeable was that barely any information was requested about the medication history or any existing medical conditions, even though certain medications can cause diarrhoea.^43^ In a Turkish study using simulated patients, the question of medication history was not asked in a single test purchase for acute diarrhoea in an adult.^27^ In a study using self‐assessment that was conducted in India, only 2% of the pharmacists surveyed indicate that they specifically ask about the medication history for acute diarrhoea in adults.^44^

Questions about the duration or the frequency of the acute diarrhoea were only asked by a little more than one‐third or about one‐fifth of the pharmacy personnel, respectively. The results are even worse in the comparable Turkish study in which these questions are only asked in 18% and 9% of all test purchases, respectively.^27^ In comparison, in the study based on self‐assessment from India, 90% of the pharmacists surveyed indicate that they specifically ask questions about the duration for acute diarrhoea in adults.^44^

Analogous to other national and international studies,^1^, ^6^, ^23^, ^24^, ^29^, ^45^ significant differences were confirmed between symptom‐based and medication‐based enquiries. In this respect, it would be important to make pharmacies or their staff more aware of the need to provide advice, particularly for medication‐based enquiries, for example, using training. Appropriate training materials are also a prerequisite for an adequate advisory service. During pharmacy studies, patient and customer consultations can be more intensively trained and evaluated using examples to refine the skills for providing advice, particularly as the teaching of ‘soft skills’ of this nature is below average in German universities to date.^46^

## Conclusions

This study was the first of its kind to investigate the quality of advice provided by pharmacies in Germany for acute diarrhoea in adults. Significant deficits were identified. Covert test purchases as part of a ‘simulated patient’ approach are a well‐tested means of revealing any weak points. However, they remain ineffective if the deficits that are identified are not communicated to the pharmacies and if the pharmacies do not subsequently implement appropriate improvement measures. Implementing the latter (comprehensively) is the key challenge facing pharmacists or their professional associations on the journey to providing better advice to customers and patients.

## Declarations

### Conflict of interest

The authors declare that they have no conflict of interests.

### Funding

This research received no specific grant from any funding agency in the public, commercial or not‐for‐profit sectors.

### Acknowledgements

The authors thank Mr. Christian Kunow, M.A. for the critical review of the manuscript and two anonymous reviewers for the very helpful comments.

### Authors’ contributions

BL conceived and designed the experiments. EB, TB, JG, MS and KS performed the experiments. BL and KS analysed the data. BL, EB, TB, JG, MS and KS wrote the manuscript. BL performed the responsibility for responding to reviewer comments. All authors state that they had complete access to the study data that support the publication.

## Supporting information


**Data S1**. Filtered assessment at pharmacy level

**Table S1**. Tests of Between‐Subjects Effects

## References

[1] Watson MC *et al* Simulated patients in the community pharmacy setting: using simulated patients to measure practice in the community pharmacy setting. Pharm World Sci 2004; 26: 32–37. 15018257 10.1023/b:phar.0000013467.61875.ce

[2] Benrimoj SI *et al* A system for monitoring quality standards in the provision of non‐prescription medicines from Australian community pharmacies. Pharm World Sci 2008; 30: 147–153. 17939057 10.1007/s11096-007-9162-7

[3] ABDA – Bundesvereinigung Deutscher Apothekerverbände . Image der Apotheken. Berlin: ABDA, 2013 http://www.abda.de/fileadmin/assets/ZDF/ZDF_2013/ZDF_2013_26-Image.pdf (accessed 11 November 2014) [in German].

[4] ABDA – Bundesvereinigung Deutscher Apothekerverbände . Die Apotheke – Zahlen, Daten, Fakten 2015. Berlin: ABDA, 2015 http://www.abda.de/uploads/tx_news/ABDA_ZDF_2013_Brosch.pdf (accessed 11 November 2014) [in German].

[5] BAH – Bundesverband der Arzneimittel‐Hersteller e.V . Der Arzneimittelmarkt in Deutschland 2014. Bonn: BAH, 2015 https://www.bah-bonn.de/index.php?eID=dumpFile&t=f&f=5526&token=8d393e946b928c6a85c98a1af98e6f2fc0fb5176 (accessed 11 November 2014) [in German].

[6] Berger K , Eickhoff C , Schulz M. Counselling quality in community pharmacies: implementation of the pseudo customer methodology in Germany. J Clin Pharm Ther 2005; 30: 45–57. 15659003 10.1111/j.1365-2710.2004.00611.x

[7] Alte D , Weitschies W , Ritter CA. Evaluation of consultation in community pharmacies with mystery shoppers. Ann Pharmacother 2007; 41: 1023–1030. 17519295 10.1345/aph.1H565

[8] Stiftung Warentest . Bittere Pillen. Test 2010; 5: 80–91 [in German].

[9] DtGV – Deutsche Gesellschaft für Verbraucherstudien mbH . Apothekenstudie 2012: Audit Servicequalität 09/2012. Berlin: DtGV, 2012 http://www.dtgv.de/wp-content/uploads/sites/4/2012/10/120910_DtGV-Test-Apothekenkooperationen_2012_kurz.pdf (accessed 11 November 2014) [in German].

[10] Stiftung Warentest . Mit Risiken und Nebenwirkungen. Test 2014; 5: 88–97 [in German].

[11] Lankisch PG *et al* Leitsymptom Diarrhö. DÄ 2006; 5: A261–A269 [in German].

[12] BAK – Bundesapothekerkammer . Arbeitshilfe der Bundesapothekerkammer zur Qualitätssicherung, Information und Beratung im Rahmen der Selbstmedikation am Beispiel der Eigendiagnose Durchfall – Anwendungsbeispiel zu den Leitlinien, Stand der Revision: 13.11.2013. Berlin: BAK, 2013 https://www.abda.de/fileadmin/assets/Praktische_Hilfen/Leitlinien/Selbstmedikation/AWB_SM_Durchfall.pdf (accessed 11 November 2014) [in German].

[13] Bruhn M . Qualitätsmanagement für Dienstleistungen – Handbuch für ein erfolgreiches Qualitätsmanagement. Grundlagen – Konzepte – Methoden, 9th edn. Berlin: Springer, 2013 [in German].

[14] Xu T , de Almeida Neto AC , Moles RJ. A systematic review of simulated‐patient methods used in community pharmacy to assess the provision of non‐prescription medicines. Int J Pharm Pract 2012; 20: 307–319. 22953770 10.1111/j.2042-7174.2012.00201.x

[15] Mesquita AR *et al* Developing communication skills in pharmacy: a systematic review of the use of simulated patient methods. Patient Educ Couns 2010; 78: 143–148. 19683890 10.1016/j.pec.2009.07.012

[16] Puspitasari HP , Aslani P , Krass I. Norris P , Granas AG. A review of counseling practices on prescription medicines in community pharmacies. Res Social Adm Pharm 2009; 5: 197–210. 19733821 10.1016/j.sapharm.2008.08.006

[17] Watson MC , Norris P , Granas AG . A systematic review of the use of simulated patients and pharmacy practice research. Int J Pharm Pract 2006; 14: 83–93. 10.1111/ijpp.12570 31397533

[18] Jesson J . Mystery shopping demystified: is it a justifiable research method? Pharm J 2004; 272: 615–617.

[19] Norris P . Reasons why mystery shopping is a useful and justifiable research method. Pharm J 2004; 272: 746–747.

[20] Stadt Neubrandenburg . Fakten & Zahlen. Neubrandenburg, 2014 http://www.neubrandenburg.de/index.php?option=com_content&view=article&id=127&Itemid=131 (accessed 11 November 2014) [in German].

[21] BMJV – Bundesministerium der Justiz und für Verbraucherschutz . Verordnung über den Betrieb von Apotheken (Apothekenbetriebsordnung – ApBetrO) § 20 Satz 1. Berlin: BMJV, 2014 http://www.gesetze-im-internet.de/apobetro_1987/BJNR005470987.html (accessed 11 November 2014) [in German].

[22] Dobbelstein T , Windbacher D . Mystery Shopping – Ziele, Prozess und Qualität eines Verfahrens zum Controlling der Dienstleistungsqualität In: Schuckel M *et al*, eds. Theoretische Fundierung und Praktische Relevanz der Handelsforschung. Wiesbaden: Dt. Univ.‐Verlag, 2007: 104–122 [in German].

[23] Horvat N , Koder M , Kos M. Using the simulated patient methodology to assess paracetamol‐related counselling for headache. PLoS ONE 2013; 7: e52510 10.1371/journal.pone.0052510. PMC3531391 23300691

[24] Puumalainen A *et al* Progress in patient counselling practices in Finnish community pharmacies. Int J Pharm Pract 2005; 13: 149–156.

[25] Mössner J . Magen‐Darm‐Mittel und Laxantien In: Schwabe U, Paffrath D, eds. Arzneiverordnungs‐Report 2013, Aktuelle Daten, Kosten, Trends und Kommentare. Berlin: Springer, 2013: 703–732 [in German].

[26] BVM – Berufsverband Deutscher Markt‐ und Sozialforscher e.V . Richtlinie für den Einsatz von Mystery Research in der Markt‐ und Sozialforschung. s.l: BVM, 2006 http://bvm.org/fileadmin/pdf/Recht_Berufskodizes/Richtlinien/RL_2006_Mystery.pdf (accessed 11 November 2014) [in German].

[27] Sancar M *et al* Assessment of the attitude of community pharmacists and pharmacy technicians towards diarrhea: a simulated patient study in Turkey. Trop J Pharm Res 2015; 14: 1509–1515.

[28] Tully MP , Beckman‐Gyllenstrand A , Bernsten CB. Factors predicting poor counselling about prescription medicines in Swedish community pharmacies. Patient Educ Couns 2011; 83: 3–6. 20605395 10.1016/j.pec.2010.04.029

[29] Kashyap KC *et al* Management of over‐the‐counter insomnia complaints in Australian community pharmacies: a standardized patient study. Int J Pharm Pract 2014; 22: 125–134. 10.1111/ijpp.12052. 23947610

[30] Alaqeel S , Abanmy NO . Counselling practices in community pharmacies in Riyadh, Saudi Arabia: a cross‐sectional study. BMC Health Serv Res 2015; 15: 557 10.1186/s12913-015-1220-6. 26669857 PMC4678714

[31] Mesquita AR *et al* Assessment of pharmacist's recommendation of non‐prescription medicines in Brazil: a simulated patient study. Int J Clin Pharm 2013; 35: 647–655. 10.1007/s11096-013-9787-7. 23740101

[32] Maher J , Hughes R . Breastfeeding guidance in community pharmacies: the results of a mystery shopper study. Nutr Diet 2013; 70: 153–157.

[33] Kashour TS *et al* Quality of assessment and counselling offered by community pharmacists and medication sale without prescription to patients presenting with acute cardiac symptoms: a simulated client study. Eur J Clin Pharmacol 2016; 72: 321–328. 10.1007/s00228-015-1981-1. 26592495

[34] Obreli‐Neto PR *et al* Use of simulated patients to evaluate combined oral contraceptive dispensing practices of community pharmacists. PLoS ONE 2013; 8: e79875 10.1371/journal.pone.0079875. 24324584 PMC3853625

[35] Hussain A , Ibrahim MI , Malik M. Assessment of disease management of insomnia at community pharmacies through simulated visits in Pakistan. Pharm Pract 2013; 11: 179–184. 10.4321/s1886-36552013000400001 PMC3869632 24367456

[36] Hussain A , Ibrahim MI . Management of diarrhoea cases by community pharmacies in 3 cities of Pakistan. East Mediterr Health J 2012; 18: 635–640. 22888622 10.26719/2012.18.6.635

[37] Van Duong D , Van Le T , Binns CW . Diarrhoea management by pharmacy staff in retail pharmacies in Hanoi, Vietnam. Int J Pharm Pract 1997; 5: 97–100.

[38] Aktekin M , Erozgen C , Donmez L. Pharmacy approach to a case of acute diarrhoea with dehydration in Antalya, Turkey. Public Health 1998; 112: 323–326. 9807929 10.1038/sj.ph.1900478

[39] Kippist C *et al* How do pharmacists respond to complaints of acute insomnia? A simulated patient study. Int J Clin Pharm 2011; 33: 237–245. 10.1007/s11096-011-9482-5. 21394573

[40] MacFarlane B , Bergin J , Peterson GM. Assessment and management of serotonin syndrome in a simulated patient study of Australian community pharmacies. Pharm Pract 2016; 14: 703 10.18549/PharmPract.2016.02.703. PMC4930858 27382424

[41] Schommer JC , Wiederholt JB . The association of prescription status, patient age, patient gender, and patient question asking behavior with the content of pharmacist‐patient communication. Pharm Res 1997; 14: 145–151. 9090700 10.1023/a:1012084207399

[42] Erku DA *et al* Extent of dispensing prescription‐only medications without a prescription in community drug retail outlets in Addis Ababa, Ethiopia: a simulated‐patient study. Drug Healthc Patient Saf 2016; 8: 65–70. 10.2147/DHPS.S106948. 27471409 PMC4948721

[43] Ratnaike RN , Jones TE . Mechanisms of drug‐induced diarrhoea in the elderly. Drugs Aging 1998; 13: 245–253. 9789728 10.2165/00002512-199813030-00007

[44] Moorthi C , Paul R , Senthilkumar C. Knowledge of community pharmacist in the management of diarrhea in adults. Der Pharm Lett 2011; 3: 364–370.

[45] Anon . Testkäufe: Gut beraten, sehr gut abgegeben, 2015 http://www.apotheke-adhoc.de/nachrichten/apothekenpraxis/nachricht-detail-apothekenpraxis/nrw-kammern-werten-15000-testkaeufe-aus/ (accessed 11 November 2014) [in German].

[46] Atkinson J . Heterogeneity of pharmacy education in Europe. Pharmacy 2014; 2: 231–243.

